# Prüfung praktisch-chirurgischer Lehre auf Distanz – Erfahrungen mit einem Hybrid-OSCE in der Chirurgie

**DOI:** 10.1007/s00104-022-01650-7

**Published:** 2022-05-20

**Authors:** S. Kurz, H. Buggenhagen, N. Wachter, L. Penzkofer, S. O. Dietz, T. T. König, M. K. Heinemann, A. Neulen, L. I. Hanke, T. Huber

**Affiliations:** 1 Rudolf Frey Lernklinik, Universitätsmedizin, Johannes Gutenberg-Universität Mainz, Mainz, Deutschland; 2 Klinik für Allgemein‑, Viszeral- und Transplantationschirurgie, Universitätsmedizin, Johannes Gutenberg-Universität Mainz, Langenbeckstr. 1, 55131 Mainz, Deutschland; 3 Zentrum für Orthopädie und Unfallchirurgie, Universitätsmedizin, Johannes Gutenberg-Universität Mainz, Mainz, Deutschland; 4 Klinik und Poliklinik für Kinderchirurgie, Universitätsmedizin, Johannes Gutenberg-Universität Mainz, Mainz, Deutschland; 5 Klinik und Poliklinik für Herz- und Gefäßchirurgie, Universitätsmedizin, Johannes Gutenberg-Universität Mainz, Mainz, Deutschland; 6 Neurochirurgische Klinik und Poliklinik, Universitätsmedizin, Johannes Gutenberg-Universität Mainz, Mainz, Deutschland

**Keywords:** Medizinische Lehre, Digitale Lehre, Digitale Prüfung, OSCE, Blended-Learning, COVID-19, Medical training, Digital teaching, Digital examination, OSCE, Blended learning, COVID-19

## Abstract

**Hintergrund:**

Die COVID-19-Pandemie hat die medizinische Lehre weltweit verändert. Digitale Lehrformate und Prüfungen konnten für kognitive Lernziele häufig gut eingesetzt werden, wohingegen praktische Fertigkeiten in Lehre und Prüfung überwiegend in Präsenz unter strengen Hygienestandards durchgeführt werden mussten.

**Ziel der Arbeit:**

Die vorliegende Untersuchung stellt die Chancen und Herausforderungen des Einsatzes eines OSCE („objective structured clinical examination“) in Präsenz und trotzdem „auf Distanz“ mit digitaler Unterstützung dar.

**Methode:**

Im Anschluss an das chirurgische Praktikum des 8. Semesters wurde eine OSCE-Prüfung in Präsenz durchgeführt, bei welcher die Studierenden in einem Raum praktische Fertigkeiten nachwiesen, während die Prüfer*innen per Videokonferenz aus einem anderen Raum zugeschaltet wurden. Die Studierenden wurden nach Abschluss der chirurgischen Lehre zur OSCE-Prüfung und zum nachhaltigen Lernen mittels standardisiertem Onlinefragebogen befragt. Zusätzlich wurden die Prüfenden zu ihren Erfahrungen befragt.

**Ergebnisse:**

Von den 157 Studierenden des Jahrgangs nahmen 25 % (*n* = 40) an der Onlinebefragung teil, wobei 36 vollständige Fragebögen ausgewertet werden konnten. Insgesamt wurde die Implementierung einer OSCE-Prüfung auch unter Pandemiebedingungen von den Studierenden als sehr positiv und sinnvoll aufgefasst (92 % der Studierenden, *n* = 33). Der Erwerb praktischer Kompetenzen wurde als sehr hoch eingeschätzt. Für 78 % (*n* = 28) der Studierenden war der Kompetenzerwerb durch die praktische Prüfung nachhaltig. Die große Mehrheit der Studierenden und Prüfenden fühlte sich durch das Hygienekonzept hinsichtlich des Infektionsschutzes sicher (92 %, *n* = 33). Insgesamt schlossen 80 Studierende (51 %) die OSCE-Prüfung nach Schulnoten (Note 1 = sehr gut, Note 6 = ungenügend) mit der Note 1, 71 Studierende (45,2 %) mit der Note 2 und 6 Studierende (3,8 %) mit der Note 3 ab.

**Schlussfolgerung:**

Praktische Prüfungen sind als Lernerfolgskontrolle psychomotorischer Lernziele unerlässlich und mit gut erarbeitetem Hygienekonzept und digitaler Unterstützung auch auf Distanz umsetzbar.

## Abstract (en)

**Background:**

The COVID-19 pandemic has changed medical teaching worldwide. Digital teaching and examinations are successful for cognitive learning objectives, whereas practical skills had to be carried out predominantly in person under strict hygiene standards.

**Aim:**

This study presents the opportunities and challenges of using a presence objective structured clinical examination (OSCE) at a distance with digital support.

**Method:**

Following surgical practical teaching an OSCE was conducted in presence, where students demonstrated practical skills in one room while the examiners were connected via videoconference from another room. Students were surveyed about the OSCE and sustained learning via a standardized online questionnaire after completion of the surgical teaching. Additionally, examiners were surveyed on their experiences.

**Results:**

In the online survey 40 students participated (25% of *n* = 157 students contacted) and 36 completed questionnaires were analyzed. Overall, the implementation of an OSCE even under pandemic conditions was perceived as very positive by the students (92% of students *n* = 33). In particular, the acquisition of practical skills was rated as very high. For 78% (*n* = 28) of the students, the acquisition of competencies through the practical examination was particularly sustainable. The vast majority of students and examiners felt safe regarding infection control because of the hygiene concept (92%, *n* = 33). Overall, 80 students achieved grade 1 (51%), 71 students grade 2 (45.2%) and 6 students grade 3 (3.8%) (grade 1 = very good, grade 6 = very bad).

**Conclusion:**

Practical examinations are essential for checking practical learning objectives and can be implemented at a distance with a well-developed hygiene concept and digital support.

## Hintergrund

Die COVID-19-Pandemie mit den daraus resultierenden Maßnahmen des Infektionsschutzgesetzes und entsprechenden Verordnungen zur sozialen Distanz, Maskenpflicht und des bundesweiten Lockdowns [1–3] bedingte eine kurzfristige Anpassung der chirurgischen Lehre sowie deren Prüfungsformate seit dem Sommersemester 2020 [4]. Bis zu diesem Zeitpunkt wurden lediglich vereinzelt digitale Formate, in erster Linie zur Unterstützung der Präsenzlehre, an der Universitätsmedizin Mainz (UMM) eingesetzt [5]. Dementsprechend war eine umfangreiche Digitalisierung der Lehre, inklusive der Prüfungsformate, zum Sommersemester 2020 erforderlich [6].

Nach Inkrafttreten der Verordnung des Bundesgesundheitsministeriums am 01.04.2020 zur Abweichung von der Approbationsordnung [7] wurden in der UMM zum einen die Vorlesungen digital aufgezeichnet und asynchron über ein Lernmanagementsystem zur Verfügung gestellt. Zum anderen wurden Seminare als Videoonlineseminare synchron virtuell abgehalten, um interaktiv mit den Studierenden arbeiten zu können. Praktika hingegen wurden in sehr kleinen Gruppen und unter strengen Hygienebedingungen durchgeführt. In diesen wurde darauf geachtet, dass die Studierenden durch E‑Learning möglichst gut vorbereitet wurden, damit der Anteil der Lehre in Präsenz auf das Notwendigste reduziert und fast ausschließlich psychomotorische Fertigkeiten vor Ort gelehrt werden konnten. Aufbau und Inhalt der Veranstaltungen orientierte sich an Lernzielen, die speziell für diese Unterrichtseinheiten bereits vor der COVID-19-Pandemie formuliert, konzertiert und mit dem Nationalen Kompetenzbasierten Lernzielkatalog Medizin (NKLM) abgeglichen wurden. Hierzu wurden die Lernziele mithilfe der Lernzielerfassungsplattform „LOOOP“ (LOOOP-Projekt, Charité Berlin, Germany) erfasst [8] und den Studierenden über Moodle^TM^ (Moodle Pty Ltd, West Perth, Australia) zur Verfügung gestellt. Prüfungen wurden häufig als Take-Home-Klausuren durchgeführt bzw. unter strengen Hygienerichtlinien in Präsenz.

Um die praktischen Lehrinhalte des Praktikums Chirurgie im Sinne des Constructive Alignments adäquat prüfen zu können, wurde entschieden, am Ende des 8. Semesters eine Objective-structured-clinical-examination(OSCE)-Prüfung in den chirurgischen Fächer durchzuführen. Das Constructive Alignment gilt als erfüllt, wenn Lernziele und Prüfungsziele abgestimmt sind und das gelehrte Kompetenzniveau (Wissenserwerb bzw. Handlungskompetenz) mit dem dafür vorgesehenen Prüfungsformat überprüft wird [9]. Zur Überprüfung kognitiver Lernziele (Wissenserwerb) eignen sich hauptsächlich schriftliche (z. B. Multiple-Choice-Klausuren) oder mündliche Prüfungen, während Handlungskompetenzen idealerweise mit praktischen Prüfungen, wie den strukturierten, standardisierten OSCE-Prüfungen, geprüft werden. Die Literatur belegt, dass OSCEs gut geeignet sind, ärztliche Fertigkeiten zu überprüfen [10, 11]. In Zeiten der Pandemie wurden auch von anderen Institutionen OSCEs unter strengen Hygieneregeln erfolgreich durchgeführt [12].

Möglichkeiten zur Durchführung hybrider OSCEs mit größtmöglichem Schutz der Teilnehmenden und Prüfenden werden in diesem Artikel gezeigt. Die Ergebnisse der studentischen Evaluation und deren Einordnung werden genutzt, um medizindidaktische Empfehlungen auszusprechen.

## Material und Methoden

Die chirurgische Lehre an der Universitätsmedizin Mainz wird gemeinsam vom Zentrum für Orthopädie und Unfallchirurgie (ZOU), der Klinik und Poliklinik für Neurochirurgie (NC), der Klinik für Herz- und Gefäßchirurgie (HG), der Klinik und Poliklinik für Kinderchirurgie (KC) sowie der Klinik für Allgemein‑, Viszeral- und Transplantationschirurgie (AVTC) organisiert. Jede der genannten Kliniken wird durch Unterrichtsbeauftragte vertreten, welche die Lernziele und Methoden des Unterrichts sowie die Prüfungen des Faches festlegen. Hierbei werden sie durch Medizindidaktiker der Rudolf Frey Lernklinik (RFLK) der Universitätsmedizin Mainz in produktivem Dialog unterstützt.

Das Wintersemester 2020/2021 wurde aufgrund der COVID-19-Pandemie und dem Fortbestehen der Abweichung von der Approbationsordnung zentral als „Hybrid-Semester“ geplant. Vorlesungen blieben weiterhin digital-asynchrone Veranstaltungen. Bei Kursen und Praktika war das Ziel, einen möglichst hohen Anteil an virtuellen Veranstaltungen – soweit sinnvoll umsetzbar – zu realisieren. Mindestens 50 % der in der Studienordnung vorgesehenen Unterrichtseinheiten für diese Kurse sollten mit praktischen Lernzielen in Präsenz stattfinden.

Zentraler Ausgang für alle Lehrveranstaltungen der Johannes Gutenberg-Universität (JGU) ist das JGU-LMS (LehrManagementSystem), welches auf Moodle^TM^ (West Perth, Australia) beruht. Moodle^TM^ ist ein Open-Source-Softwarepaket, welches die Konzeption und Durchführung onlinebasierter Lehre unterstützt. Durch eine Arbeitsgruppe des Kompetenzteams „Digitale Lehre“ der JGU wird das JGU-LMS stets den entsprechenden Bedürfnissen der Lehrenden angepasst. Außerdem werden diese didaktisch und technisch durch vielzählige Schulungsangebote unterstützt.

Die Vorlesung der chirurgischen Lehre für das 8. Semester wurde mithilfe der Software Panopto© (Panopto, Inc, Seattle, WA, USA) in für Videotechnik ausgestatteten Räumlichkeiten der RFLK durch die Lehrenden aufgezeichnet und über einen personenbezogenen geschützten Zugang auf das JGU-LMS den Studierenden verfügbar gemacht. Die Vorlesungen konnten jederzeit asynchron angesehen werden.

Das Praktikum Chirurgie wird im 8. Semester traditionell als Blockveranstaltung am Anfang des Semesters durchgeführt. Aufgrund der pandemiebedingten Hygienevorschriften wurde das Praktikum mit 157 Studierenden in halbierter Gruppengröße und mit reduzierten Themen (5 statt 10 Praktikumsstationen) durchgeführt. Zusätzlich wurden Lehrvideos, unter anderem zu Nahttechniken, abdomineller Untersuchung, Schultergelenkuntersuchung und zur hygienischen Händedesinfektion, durch die chirurgischen Kliniken in Kooperation mit der RFLK produziert und den Studierenden online über JGU-LMS zur Verfügung gestellt. Ein unter normalen Umständen vorgesehenes ergänzendes Training im Skills Lab als Vorbereitung auf die OSCE-Prüfung musste aufgrund erneut steigender Infektionszahlen in ein digitales, interaktives Training umgewandelt werden. Für die praktische Übung chirurgischer Nähte wurde ein „Take-Home“-Set, bestehend aus Nadelhalter, Pinzette und Nahtmaterial, kostenfrei zur Verfügung gestellt. An 6 zusätzlichen Terminen konnte dieses Thema im Sinne eines interaktiven Tutorials als Videokonferenz vertieft werden.

Das Praktikum des 8. Semester wurde im Wintersemester 2020/2021 erstmalig als OSCE unter einem streng ausgearbeiteten Hygienekonzept geprüft. An 5 Stationen sollten die Studierenden ihre im Kurs erlernten praktischen Fertigkeiten zeigen. Der Parcours griff die wesentlichen gelehrten chirurgischen Fertigkeiten auf. Zu diesen gehörten die Hautnaht, thorakale Zugangswege, Anwendung der Glasgow Coma Scale, abdominelle Untersuchung und chirurgische Händedesinfektion. Pro Station konnten max. 20 Punkte erzielt werden. An einer 6. Station wurden 5 Theoriefragen gestellt, die durch Nutzung der Software Ilias (ILIAS open source e‑Learning, e. V., Köln, Deutschland) an einem Computer zu beantworten waren. Da pro Frage 2 Punkte (max. 10 insgesamt) erreicht werden konnten, war die Gewichtung der Theorie somit geringer. Die maximal zu erreichende Gesamtpunktzahl der chirurgischen Prüfung betrug 110 Punkte. Alle Checklisten wurden in einem Reviewprozess inhaltlich und praktisch überprüft. Die OSCE-Checklisten waren in „Thema der Station“, „Prüfungsziele“, „Fallvignette“, „Aufgabenstellung“ und „Bewertung“ unterteilt. Bis auf das Bewertungsschema wurden diese den Studierenden zu Beginn der jeweiligen Station standardisiert schriftlich zur Verfügung gestellt. Das Verständnis der Aufgabenstellung wurde durch den Prüfer zu Beginn abgefragt.

Bewertungskriterien für „Regelgerechte Durchführung von 2 Einzelknopfnähten am Modell“:

- regelgerechtes Einspannen der Nadel in den Nadelhalter,
- regelgerechtes Führen der Pinzette,
- korrektes Weichteilmanagement (Haut wird nicht traumatisiert),
- regelgerechtes Führen des Nadelhalters,
- korrektes Abwerfen der Nadel in den Abwurf,
- regelgerechter Abstand von Einstich und Wundrand (0,5–1 cm),
- korrekte Durchführung eines Instrumentenknotens,
- Nahtergebnis (gut adaptierte Wundränder, Stichabstand 0,5–1 cm).

Die genannten Kriterien wurden jeweils mit Punkten bewertet, wobei die Gewichtung durch die chirurgischen Unterrichtsbeauftragten erfolgt. Die Gesamtpunktzahl lag bei 20 Punkten.

Die Prüfenden wurden standardisiert geschult und ihnen wurden Instruktionen zu den jeweiligen Aufgaben gegeben. Die Objektivität der Prüfung wurde durch die Abstimmung der Beurteilenden in einem vorangehenden Training und nachfolgender Erstellung präziser Beurteilungskriterien innerhalb eines Beurteilungsbogens gewährleistet. Hierbei wurde „regelgerecht“ und „korrekt“ jeweils festgelegt und ggf. mit Abbildungen oder Videos zur Prüferschulung standardisiert.

**Figure 1:**
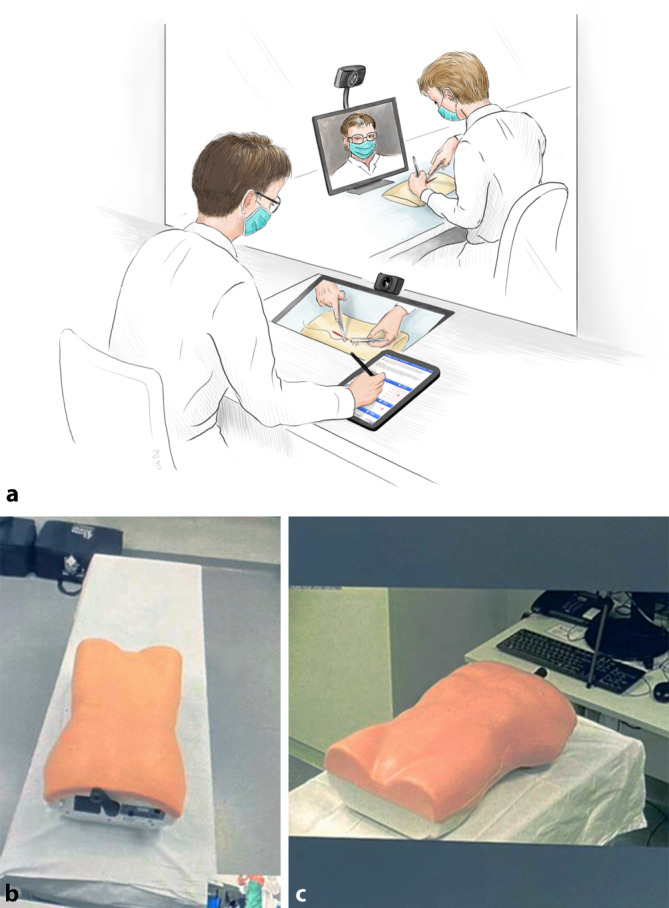

Die Durchführung fand hybrid als „OSCE-Prüfung auf Distanz“ statt. Die Studierenden sowie die Prüfer kamen zur Prüfung in die RFLK, waren aber räumlich vom Prüfer bzw. der Prüferin getrennt. Beide kommunizierten über ein bilaterales Videokonferenzsystem (MS Teams, Microsoft Corporation, Redmond, WA, USA). Zusätzlich konnten die Prüfer*innen die Studierenden durch eine verspiegelte Scheibe sehen (Abb. 1). In den Räumlichkeiten wurde auf entsprechende Einhaltung der Hygienevorschriften der UMM geachtet (Maskenpflicht, regelmäßiges Lüften, Oberflächendesinfektion, Tragen von Handschuhen).

Zur Erhebung der Einstellungen und Erfahrungen mit der „OSCE-Prüfung auf Distanz“ im Wintersemester 2020/2021 wurden zur Durchführung einer Querschnittsstudie Fragebögen für Studierende der Universitätsmedizin Mainz entwickelt. Die Erstellung und Durchführung erfolgten online über LimeSurvey© (LimeSurvey GmbH, Hamburg, Deutschland), welches über die JGU lizenziert ist. Die Teilnahme an der Befragung war anonym und freiwillig möglich. Die Einladung zur Befragung erhielten die Studierenden im darauffolgenden Semester per E‑Mail. Der Fragebogen beinhaltete insgesamt 13 Fragen, wovon 12 mit Auswahlfunktion und eine mit Freitextfeld versehen waren. Der Link zur Befragung wurde am 17.08.2021 per E‑Mail versendet, am 26.08.2021 wurde erneut per E‑Mail an die Teilnahme erinnert. Weiterhin wurden die Dozierenden der OSCE-Prüfungen des Wintersemester 2020/2021 und des Sommersemesters 2021 zu ihren Erfahrungen (Hygienekonzept, Prüfungsinhalte, Vorbereitungsaufwand, praktische Umsetzung) mit einem kurzen Onlinefragebogen ebenfalls über LimeSurvey© (LimeSurvey GmbH, Hamburg, Deutschland) befragt.

## Ergebnisse

An der im August 2021 stattgefunden Onlinebefragung nahmen 40 Studierende teil (25 % bei *n* = 157 angeschriebenen Studierenden). 38 Studierende füllten die Befragung vollständig aus, hiervon waren 24 Studierende weiblich und 14 männlich. Die Studierenden waren im Mittel 27,0 Jahre alt, wobei einmal keine Angabe erfolgte. Letztendlich konnten 36 Fragebögen vollständig ausgewertet werden. Zwei Dropouts kamen zustande, da diese Studierenden angaben, nicht am OSCE teilgenommen zu haben.

**Figure 2:**
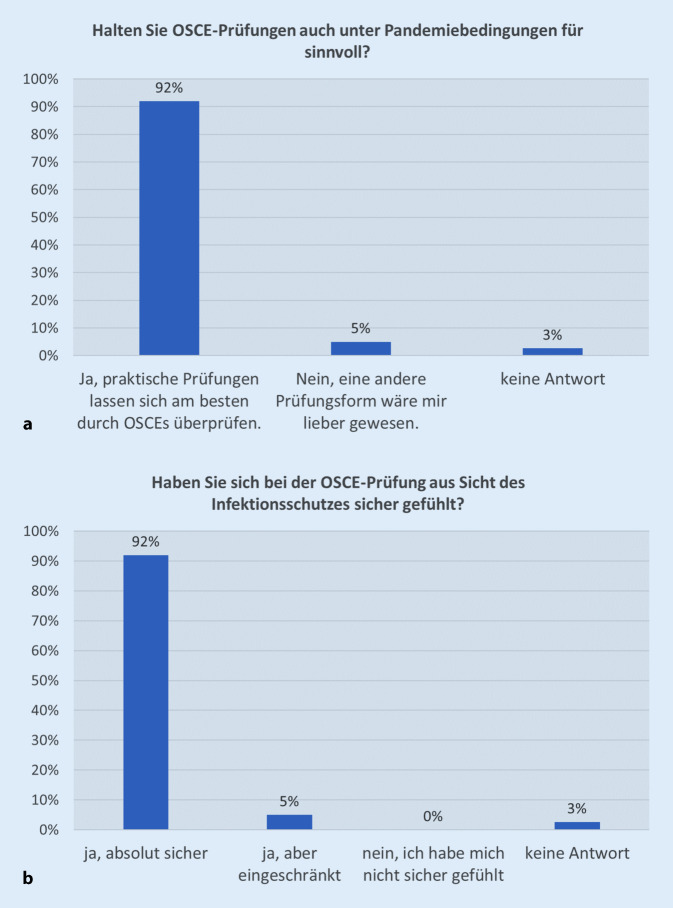

Auch unter Pandemiebedingungen halten 92 % der Studierenden OSCE-Prüfungen für sinnvoll, lediglich 2 Studierende hätten sich eine andere Prüfungsform gewünscht und ein Teilnehmender traf keine Aussage (Abb. 2a). Aus Sicht des Infektionsschutzes fühlten sich 92 % der Befragten bei der OSCE-Prüfung absolut. Ein Studierender beantwortete die Frage nicht und 2 Teilnehmende fühlten sich eingeschränkt sicher (Abb. 2b).

Dass eine OSCE-Prüfung zum Lernen motiviert und sie ohne diese Prüfungsform die praktischen Lernziele weniger nachhaltig gelernt hätten, sagten 78 % der Befragten. Vier Studierende gaben an, dass sie auch ohne OSCE die praktischen Fertigkeiten des chirurgischen Praktikums des 8. Semesters nachhaltig gelernt hätten. Vier Studierende machten hierzu keine Aussage.

**Figure 3:**
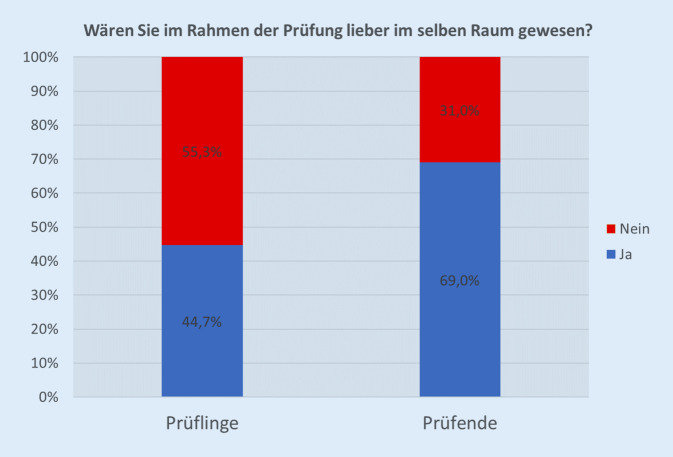

Die Einschätzung der Distanz zwischen Prüfenden und Prüflingen zeigt Abb. 3.

Für 86 % der Studierenden funktionierte die Kommunikation trotz der räumlichen Trennung technisch sehr gut, 11 % berichteten, dass die Kommunikation aufgrund technischer Störungen nicht optimal funktioniert hat, einmal erfolgte keine Angabe.

Eine größere Distanz zwischen Teilnehmenden und Prüfenden können sich 13 % der Studierenden vorstellen. Für 69 % war die Distanzierung wie im OSCE im WS 20/21 genau richtig, eine größere Distanz (Prüfung an verschiedenen Standorten z. B. von zu Hause aus) wurde nicht gewünscht. Weitere 11 % lehnen eine räumliche Trennung von Teilnehmenden und Prüfenden grundsätzlich ab. Ein Teilnehmender machte hierzu keine Angaben. Tab. 1 zeigt die Selbsteinschätzung verschiedener praktischer chirurgischer Fertigkeiten nach Abschluss der kompletten chirurgischen Lehre.

**Table 1:** 

Kompetenz	Kompetenzeinschätzung in Schulnoten MW [95 %-CI]
Korrekte Durchführung der chirurgischen Händedesinfektion	1,42 [1,224–1,618]
Klinische Untersuchung des Abdomens	1,39 [1,199–1,590]
Durchführung einer Einzelknopfnaht	1,50 [1,287–1,713]
Anwendung der Glasgow Coma Scale	1,71 [1,458–1,963]
Demonstration thorakaler operativer Zugangswege mit Vor- und Nachteilen	2,26 [1,933–2,593]

Die Studierenden bewerteten die OSCE-Prüfung mit der Schulnote 1,9. Im Vergleich erreichte das Praktikum im Wintersemester 20/21 nach Abschluss eine Gesamtnote von 2,4. Die Möglichkeit des selbstständigen Übens mit den zur Verfügung gestellten Nahtsets sowie das Online-Naht-Tutorium wurden sehr positiv aufgenommen, 48 Studierende nahmen an der freiwilligen Veranstaltung teil (28,6 %).

Insgesamt schlossen 80 Studierende (51 %) die OSCE-Prüfung mit „sehr gut“, 71 Studierende (45,2 %) mit „gut“ und 6 Studierende (3,8 %) mit „befriedigend“ ab.

An der Befragung der Dozierenden nahmen 16 von 26 Dozierenden teil (62 %). Alle teilnehmenden Prüfer hielten die Durchführung einer OSCE-Prüfung unter den angewendeten Hygienemaßnahmen für vertretbar und 81 % hielten diese hinsichtlich der Prüfung praktischer Fertigkeiten für sinnvoll. Die Anwendung der Tablets zur Prüfungsbewertung wurde gut angenommen und als einfach und intuitiv bewertet (trifft zu *n* = 11, 69 %; trifft eher zu *n* = 5, 31 %). Die Durchführung und Vorbereitung der OSCE-Prüfung wurde als eher aufwendig empfunden. So gaben lediglich 5 Teilnehmer (31 %) an, sich weniger als 10 min auf die Prüfung vorbereitet zu haben, 7 (44 %) brachten 10–30 min für die Vorbereitung auf und 4 (25 %) sogar mehr als 30 min. Dem entsprechend wurde die Vereinbarkeit mit dem klinischen Alltag von 56 % der Teilnehmer als gegeben gesehen, wobei 44 % eher unentschlossen waren.

## Diskussion

Die OSCE-Prüfung wurde auch unter Pandemiebedingungen gut bewertet und wird von Lehrenden und Studierenden als sinnvoll erachtet. Bis auf 2 Personen fühlten sich alle Studierenden bezüglich des Infektionsschutzes sehr sicher. Zum Zeitpunkt der Durchführung waren Antigentests auf SARS-CoV‑2 noch nicht flächendeckend für Studierende eingeführt, aber auch ohne diese gelang es, die Prüfung weitestgehend sicher durchzuführen. Ein Teil der Prüfer hatte bereits aufgrund der ärztlichen Tätigkeit die 1. oder bereits die 2. COVID-Impfungen erhalten. Ebenso wurden bereits einige Studierende ein- oder zweimal aufgrund ihrer studentischen oder beruflichen Tätigkeiten geimpft. Es musste allerdings davon ausgegangen werden, dass es Personen gab, die noch keinen vollständigen Impfschutz hatten, sodass die Distanz zwischen Studierenden und Prüfern möglichst gewährleistet werden sollte. Dies gelang durch die Videoübertragung mit gleichzeitiger räumlicher Trennung sehr gut. Eine Abfrage des Impfstatus wurde aus datenschutzrechtlichen Gründen nicht durchgeführt.

Durch die Hygieneauflagen mussten sowohl Lehrstationen (5 statt 10) als auch Prüfungsstationen (5 praktische und eine theoretische Station statt 8 praktischen Stationen) reduziert werden, dadurch waren die Gelegenheiten zum praktischen Üben eingeschränkt. Die Kompensation durch Lehrvideos verbesserte die Situation, reichte aber nicht vollumfänglich aus, da das aktive Handeln fehlte.

Die räumliche Trennung von den Prüfenden wurde von den Studierenden uneinheitlich bewertet. Etwa die Hälfte befürwortete die Trennung, während die andere Hälfte sich keine räumliche Trennung wünschte. Die unterschiedliche Beurteilung könnte abhängig vom Impfstatus bzw. der persönlichen Einschätzung der Pandemie gewesen sein. Die Kommunikation per Video funktionierte in den überwiegenden Fällen sehr gut. In Einzelfällen gab es technische Schwierigkeiten bei der Videoübertragung, sodass nur die Sprache übertragen werden konnte, was berechtigterweise bemängelt wurde. Im Freitext wurde einmal erwähnt, dass die Trennung von Studierenden und Prüfenden die Nervosität beim OSCE erhöht hätte. Obwohl ein Großteil der Lehre zum Zeitpunkt der Prüfung seit ca. 10 Monaten von zu Hause aus stattfand, konnten sich nur 13 % der Befragten eine größere Distanz, z. B. mit Prüfenden oder Teilnehmenden von zu Hause aus, vorstellen.

Analog zur Literatur gab ein Großteil der Studierenden an, durch die OSCE-Prüfung motiviert zu sein, praktische Lernziele zu erlernen, bzw. dass sie ohne die Prüfung weniger nachhaltig gelernt hätten.

Im Freitext wurden die sehr gute Organisation sowie die klaren Strukturen durch das Vorbereitungsmaterial und die Lernziele gelobt. Präsenzunterricht wurde von allen Teilnehmenden als essenziell zum Erlernen psychomotorischer Lerninhalte gesehen. Dies beinhaltet sowohl die Interaktion mit Patienten als auch das Vermitteln praktischer Fähigkeiten, wie chirurgisches Nähen oder die chirurgische Händedesinfektion. Viele Lehrinhalte sollten aus Sicht der Studierenden bereits in früheren Semestern gelehrt werden, da diese Skills sonst bereits in Famulaturen gefordert bzw. gelernt würden.

Die Akzeptanz durch die Prüfenden war insgesamt gut und das Engagement und der Einsatz groß. Trotz erhöhtem Zeitaufwand und Zusatzbelastung im klinischen Alltag wurde die Durchführung der OSCE-Prüfung als sinnvoll und hygienisch einwandfrei erachtet.

OSCE-Prüfungen gehen mit einem großen technischen und personellen Aufwand einher, was in Zeiten strenger Budgetierung kritisch reflektiert werden muss. Im Vergleich zu sonst (unter Nichtpandemiebedingungen) durchgeführten OSCEs an der Universitätsmedizin Mainz war eine weitere studentische Hilfskraft zur technischen Unterstützung sowie 2 statt ein Tutor zur Koordination und Zwischendesinfektion notwendig. Dieser zusätzliche personelle Aufwand scheint nach Auswertung der vorliegenden Daten gerechtfertigt, um eine praktische Prüfung zu ermöglichen. Die Lernziele konnten adäquat geprüft werden und gerade bei eingeschränkter Präsenzlehre geben OCSEs sowohl Studierenden als auch Lehrenden eine gute Rückmeldung über den Kompetenzgewinn praktischer Lernziele. International wurden im Rahmen der COVID-19-Pandemie virtuelle OSCE-Prüfungen (VOSCE), elektronische OSCE (eOSCE) und ähnliche Varianten beschrieben [13, 14]. Diese beinhalten die standardisierte Prüfung durch Videoübertragung (z. B. Zoom®) an standardisierten Fallbeispielen. Für die Chirurgie erscheint eine praktische Prüfung der Nahttechniken, der Händedesinfektion oder der abdominellen Untersuchung am Phantom jedoch nur schwer in reinen Fallbeispielen prüfbar. Unseres Wissens gibt es keinen beschriebenen praktischen OSCE unter Pandemiebedingungen in der Literatur, sodass der beschriebene Hybrid-OSCE auf Distanz als Beispiel für digitale Lehre auch für psychomotorische Fertigkeiten Vorbildcharakter entwickeln könnte.

Der aktuelle Referentenentwurf zur Änderung der ärztlichen Approbationsordnung sieht ebenfalls eine verstärkte Implementierung der digitalen Lehre vor [15]. Zukünftig sollen digitale Lehre und Präsenzlehre im Sinne von Blended-Learning-Konzepten vereint werden. Beim Blended-Learning werden Onlinelernelemente mit Präsenzformaten verknüpft, bei denen das vorab online erlernte Wissen in praktischen Einheiten durch Erfahrungsaustausch oder Rollenspiele angewendet und gefestigt werden soll [16]. Beim Aufbau der Lehreinheiten ist es sinnvoll, zunächst die Kompetenzen an Simulatoren bzw. mit Simulationspatienten zu trainieren, bevor die Studierenden zum Bedside-Teaching übergehen [17]. Dies ist hier durch die Splittung in Semester 8 (überwiegend praktische Fertigkeiten in Simulation) und Semester 9 (überwiegend Patientenkontakt) berücksichtigt. Auch wenn gezeigt werden konnte, dass psychomotorische Fähigkeiten von Studierenden durch qualitative Onlinelehrmodule gut geschult werden können [18], sollte für die Vermittlung praktischer Fertigkeiten weiterhin hauptsächlich Präsenzunterricht eingeplant werden, da der zwischenmenschliche Kontakt sowohl mit Lehrenden als auch mit Patienten unerlässlich ist [19–21]. Voraussichtlich werden diese Lehrformate zukünftig durch digitale Unterstützungsverfahren wie virtuelle oder augmentierte Realität und unter Anwendung von künstlicher Intelligenz weiter ergänzt, was für Lehrende und Studierende weitere Umstellungen im Umgang mit Lerninhalten und Unterrichtsveranstaltungen bedeutet [22]. Auch werden in der Prüfungsvorbereitung zukünftig Lehreinheiten durch Gamification-Learning ergänzt werden [23]. Mit Blick auf die neue Approbationsordnung werden OSCEs und Vorbereitungen auf diese Prüfungsform in Zukunft eine große Rolle spielen, allerdings auch mit hohem personellem Aufwand und Kosten verbunden sein, sodass moderne digitale Unterstützungssysteme diesbezüglich kontinuierlich evaluiert werden sollten.

Insgesamt wurden die pandemiebedingten Anpassungen des chirurgischen Praktikums durch die Studierenden gut angenommen.

Durch das hohe Engagement der Lehrenden, ergänzt durch studentische Tutoren, konnte eine hochwertige Lehre und praktische Prüfung gewährleistet werden. Dies ist ein enorm wichtiger Faktor bei der Ausbildung des zukünftigen ärztlichen Personals unter den – andauernden – Einschränkungen der Präsenzlehre.
